# LncRNA H19 promotes triple-negative breast cancer cells invasion and metastasis through the p53/TNFAIP8 pathway

**DOI:** 10.1186/s12935-020-01261-4

**Published:** 2020-05-29

**Authors:** Yang Li, Hong-Yu Ma, Xiao-Wei Hu, Yuan-Yuan Qu, Xin Wen, Yu Zhang, Qing-Yong Xu

**Affiliations:** 1 Department of Breast Radiotherapy, Harbin Medical University Cancer Hospital, No.150 Haping Road, Nangang District, Harbin, 150081 Heilongjiang People’s Republic of China; 2 Department of Head and Neck and Genito-Urinary Oncology, Harbin Medical University Cancer Hospital, Harbin, 150081 People’s Republic of China; 3 Department of Ultrasound, Harbin Medical University Cancer Hospital, Harbin, 150081 People’s Republic of China

**Keywords:** lncRNA H19, Breast cancer, Metastasis, p53, TNFAIP8

## Abstract

**Background:**

Long non-coding RNA H19 (lncRNA H19) has been implicated in tumorigenesis and metastasis of breast cancer through regulating epithelial to mesenchymal transition (EMT); however, the underlying mechanisms remain elusive.

**Methods:**

LncRNA H19 and TNFAIP8 were identified by qRT-PCR and western blotting. CCK-8 assay, clone formation assay, transwell assay, and flow cytometry assay were performed to determine cell proliferation, migration, invasion and cell cycle of breast cancer respectively. Western blotting and immunohistochemistry (IHC) were utilized to evaluate the protein expression levels of p53, TNFAIP8, and marker proteins of EMT cascades in vivo. Dual luciferase reporter assay and RNA pull down assay were conducted to evaluate the interactions of lncRNA H19, p53 and TNFAIP8.

**Results:**

The expression of lncRNA H19 and TNFAIP8 was up-regulated in breast cancer tissues and cell lines, especially in triple-negative breast cancer (TNBC). Functionally, knockdown of lncRNA H19 or TNFAIP8 coused the capacities of cell proliferation, migration, and invasion were suppressed, and cell cycle arrest was induced, as well as that the EMT markers were expressed abnormal. Mechanistically, lncRNA H19 antagonized p53 and increased expression of its target gene TNFAIP8 to promote EMT process. Furthermore, silencing of lncRNA H19 or TNFAIP8 also could inhibit tumorigenesis and lymph node metastases of MDA-MB-231 cells in xenograft nude mouse models.

**Conclusions:**

Our findings provide insight into a novel mechanism of lncRNA H19 in tumorigenesis and metastases of breast cancer and demonstrate H19/p53/TNFAIP8 axis as a promising therapeutic target for breast cancer, especially for TNBC.

## Background

Breast cancer is the most commonly diagnosed cancer and the leading cause of cancer-related death among females worldwide [1]. It is a heterogeneous group of neoplasms with at least three subtypes, each of which has different response to treatment and preferential organ sites of metastases [2, 3]. Triple-negative breast cancer (TNBC), one subtype of breast cancer with a negative expression profile for the three proteins, estrogen receptor (ER), progesterone receptor (PR) and human epidermal growth factor receptor type 2 (HER2), constitutes about 15% of breast cancer cases [4] and has high risk of metastasis [5], particularly to the visceral organs, such as the lung, liver, and brain. The median overall survival is less than 1 year for women with metastatic TNBC, and almost all die of this disease regardless of intensive and toxic systemic chemotherapy [6]. Thus, developing new therapeutic interventions for preventing and treating metastasis in TNBC patients is one of the highest priorities of current breast cancer research.

Over the last few decades, a variety of long non-coding RNAs (lncRNAs) have been implicated in breast cancer development [7]. In this context, lncRNA H19 might be of special interest, as it is considered both a driving force of tumorigenesis and metastasis of breast cancer [8, 9]. LncRNA H19 has been implicated in regulating the transition between epithelial and mesenchymal cells (EMT/MET switch) [10], making it an essential inducer of early metastatic events. Recent studies have showed that lncRNA H19 operates as an oncogene by functioning as a myc up-regulated gene [11], a ceRNA sponge [12], or a precursor of miR-675 [13]. Nevertheless, the mechanisms are still not fully understood hereinafter we speculate that lncRNA H19 may regulate HCC progression through some other ways.

The tumor suppressor p53 is critical for the maintenance of genome stability and the prevention of tumor formation [14, 15]. As the most frequently mutated gene in human tumors, the frequency of p53 mutation differs among the different breast cancer subtypes, with TNBC having the highest prevalence of 80% [16]. Importantly, p53 negatively regulates lncRNA H19 in tumor cells [17]. To facilitate G1/S transition in the cell cycle, lncRNA H19 needs to escape from repression by p53 that primarily triggers G1/S and G2/M cell cycle arrest [18, 19]. Moreover, lncRNA H19 can interact with p53 protein, causing its partial inactivation in gastric- and bladder cancer cells [20, 21]. However, the regulation of p53 by lncRNA H19 in human breast cancer remains largely unexplored.

Increasing evidence shows that tumor necrosis factor-α-induced protein 8 (TNFAIP8), which are produced by TNFα, is overexpressed in various human cancers and play crucial mechanistic roles in cell survival, proliferation, and metastasis [22–24]. Importantly, TNFAIP8 was closely linked to axillary lymphatic metastasis and was identified as an independent prognostic factor for TNBC patients [25]. Moreover, TNFAIP8 variant 2 has been identified as a p53-regulated gene product that exerts tumor-promoting effects through reciprocal regulatory interactions with p53, and depletion of TNFAIP8 variant 2 in MCF-7 cells was found to induce G1 or G2/M arrest [26]. On the other hand, whether lncRNA H19 is involved in the p53-TNFAIP8 interplay in breast cancer, especially in TNBC, remains to be determined.

In the current study, we found that lncRNA H19 and TNFAIP8 were markedly enhanced in breast cancer tissues and cell lines, with TNBS cells in particular. In addition, lncRNA H19 altered the expression of cascades of EMT makers and promoted proliferation, migration and invasion of breast cancer cells through positive regulation of TNFAIP8. Mechanically, lncRNA H19 functioned as a competitive inhibitor of p53 and abrogated its transcriptional inhibition of TNFAIP8. Our study provides the first evidence for the interaction of lncRNA H19, p53, and TNFPI8 in breast cancer cell progression and metastases, highlighting the promising therapeutic potential of this axis for treatment of breast cancer.

## Methods

### Tissue samples

Sixty patients (40–73 years old, women) who were pathologically diagnosed as TNBC (Grade 2 or 3 infiltrating ductal carcinoma with negative expression of ER, PR and HER2 proteins, or accompanied by medullary features, infiltrating micropapillary carcinoma, or occasional vascularized thrombus) and non-TNBC (Grade 2 or 3 infiltrating ductal carcinoma with not all negative expression of ER, PR and HER2 proteins) and did not undergo chemotherapy or radiotherapy before surgery were enrolled in this study. The clinicopathological data was showed in Table 1. Tumor tissue samples and adjacent non-tumor tissue specimens were collected and immediately frozen in liquid nitrogen at −80 °C. The study was approved by the Ethics Committee of Harbin Medical University Cancer Hospital, with informed consent obtained from all patients.

**Table 1:** The clinicopathological data of clinical tumor tissue samples

Group	Sample number	HER2	PR	ER	Age of patients	Pathological grading
TNBC	HMU31500600J07	(−)	(−)	(−)	51	T1N0M0 I
	HMU31500574J07	(−)	(−)	(−)	73	T1N0M0 I
	HMU31500519J07	(−)	(−)	(−)	64	T1N0M0 I
	HMU31500310J07	(−)	(−)	(−)	61	T1N0M0 I
	HMU31500306J07	(−)	(−)	(−)	61	T1N0M0 I
	HMU31500294J07	(−)	(−)	(−)	52	T1N0M0 I
	HMU31500268J07	(−)	(−)	(−)	61	T1N0M0 I
	HMU31500492J07	(−)	(−)	(−)	77	T2N0M0 IIA
	HMU31500473J07	(−)	(−)	(−)	61	T2N0M0 IIA
	HMU31500376J07	(−)	(−)	(−)	40	T2N0M0 IIA
	HMU31500350J07	(−)	(−)	(−)	45	T2N0M0 IIA
	HMU31500563J07	(−)	(−)	(−)	52	T2N0M0 IIA
	HMU31500552J07	(−)	(−)	(−)	64	T2N0M0 IIA
	HMU31500529J07	(−)	(−)	(−)	65	T2N0M0 IIA
	HMU31500171J07	(−)	(−)	(−)	63	T2N1M0 IIB
	HMU31500193J07	(−)	(−)	(−)	56	T2N1M0 IIB
	HMU31500370J07	(−)	(−)	(−)	65	T2N1M0 IIB
	HMU31500213J07	(−)	(−)	(−)	43	T2N1M0 IIB
	HMU31500338J07	(−)	(−)	(−)	49	T2N1M0 IIB
	HMU31500150J07	(−)	(−)	(−)	51	T2N1M0 IIB
Non-TNBC	HMU31500700J07	(3+)	(−)	(−)	56	T1N0M0 I
	HMU31500698J07	(3+)	(−)	(−)	61	T1N1M0 IIA
	HMU31500636J07	(3+)	(−)	(−)	51	T1N0M0 I
	HMU31500710J07	(3+)	(−)	(−)	49	T1N0M0 I
	HMU31500731J07	(3+)	(−)	(−)	53	T1N1M0 IIA
	HMU31500688J07	(2+)	(+)	(+)	70	T1N0M0 I
	HMU31500684J07	(2+)	(+)	(+)	58	T1N0M0 I
	HMU31500666J07	(+)	(+)	(+)	66	T1N0M0 I
	HMU31500697J07	(+)	(+)	(+)	62	T1N1M0 IIA
	HMU31500680J07	(2+)	(+)	(+)	50	T1N1M0 IIA
	HMU31500702J07	(−)	(+)	(+)	43	T2N1M0 IIB
	HMU31500687J07	(2+)	(+)	(+)	52	T2N1M0 IIB
	HMU31500631J07	(2+)	(+)	(+)	50	T2N1M0 IIB
	HMU31500627J07	(−)	(+)	(+)	48	T2N1M0 IIB
	HMU31500732J07	(2+)	(+)	(+)	57	T2N1M0 IIB
	HMU31500755J07	(3+)	(+)	(+)	64	T2N1M0 IIB
	HMU31500787J07	(3+)	(+)	(+)	53	T1N1M0 IIA
	HMU31500884J07	(−)	(+)	(+)	44	T1N1M0 IIA
	HMU31500829J07	(+)	(+)	(+)	52	T1N1M0 IIA
	HMU31500817J07	(2+)	(+)	(+)	65	T1N1M0 IIA

### Cell culture

The human breast epithelial cell line MCF10A and breast cancer cell lines MCF-7, SK-BR-3, BT-549, MDA-MB-231and MDA-MB-468 were purchased from the American Type Culture Collection (Manassas, VA, USA). All cells were maintained at 37 °C with 5% CO_2_ and cultured in Dulbecco’s modified Eagle’s medium (DMEM; Thermo Fisher Scientific, MA, USA) supplemented with 10% fetal bovine serum (FBS; Thermo Fisher) and 1% penicillin–streptomycin (Invitrogen, CA, USA).

### Cell transfection

Specific H19 siRNAs, p53 siRNAs, TNFAIP8 siRNAs and a scrambled locus siRNA (NC) were designed and synthesized by GenePharma (Shanghai, China). The 3′UTRs of TNFAIP8 were amplified by PCR from the genomic DNA of BT-549 and MDA-MB-231 cells and were cloned into pcDNA3.1(+) according to standard protocols. BT-549 and MDA-MB-231 were transfected with siRNA control (si-control), siRNA H19 (si-H19), p53-siRNAs and TNFAIP8-siRNAs using lipofectamine 3000 (Invitrogen, Eugene, OR, USA). For plasmid transfection, the plasmids of pcDNA3.1(+)-control, pcDA3.1(+)-H19, and pcDNA3.1(+)-TNFAIP8 were transfected into cells with the X-treme GENE HP DNA Transfection Reagent (Sigma-Aldrich, St. Louis, MO, USA). 24 h after transfection, cells were harvested for the subsequent experiments.

### RNA extraction and quantitative real-time polymerase chain reaction (qRT-PCR)

Total RNA was purified from breast cancer cells and tissues using TRIzol reagent (Invitrogen, Shanghai, China). For miRNA expression detection, complementary DNA (cDNA) was synthesized using the MystiCq^®^ microRNA cDNA Synthesis Mix Kit (Sigma-Aldrich) according to the manufacturer’s instruction. Quantitative PCR was performed using SuperScript™ III Platinum™ One-Step qRT-PCR Kit (Thermo Fisher). For the quantification of lncRNA and TNFAIP8, RNA was subjected to two-step cDNA synthesis using PrimeScript™ RT reagent Kit (Takara Biomedical Technology, Dalian, China). qRT-PCR was performed using a standard SYBR Green Premix Ex Taq Kit (Takara Biomedical Technology, Dalian, China) on an ABI7300 thermal cycler (Applied Biosystem, Shanghai, China). A 2^−∆∆Ct^ method was employed to assess the relative expression levels of genes. GAPDH was chosen as the reference gene for lncRNA and mRNA, and U6 was the internal reference gene for miRNA.

### Cell viability assay

Cell viability was determined by a Cell Counting Kit-8 (CCK-8) (Sangon, Shanghai, China). In brief, 7 × 10^3^ cells were cultured in each 96-well plate, and incubated for 24, 48, 72, or 96 h, respectively. Subsequently, 10% CCK-8 reagent was added to each well for an additional 1 h before the endpoint of incubation at 37 °C in dark. The absorbance was measured at 450 nm (A_450_) by a microplate reader (Thermo Fisher). Experiments were repeated at least three times in 6 replicate wells per sample.

### Colony formation assay

After transfection treatment for 24 h, BT-549 and MDA-MB-231 cells were seeded in 6-well plates (300 cells/well) and incubated for 10 days in DMEM supplemented with 10% FBS. The plates were then washed three times after fixation with 2.5% glutaraldehyde. Fixed cells were subsequently stained with 2% Giemsa stain for 60 min at room temperature. Numbers of colonies (with > 50 cells/colony) were counted under a light microscopy.

### Transwell migration and invasion assay

After transfection treatment for 24 h, BT-549 and MDA-MB-231 cells (2 × 10^4^) were resuspended in 200 μl serum-free DMEM medium and seeded into the upper transwell chambers (Corning, NY, USA) containing 8 μm pores. Culture medium supplemented with 10% FBS was added to the lower chamber. After incubation for 14 h for migration and 20 h for invasion at 37 °C, the cells on the upper surface were wiped off and cells on the lower surface were stained with 0.1% crystal violet. The average number of migrated and invasive cells were counted and photographed under a light microscopy (×200) (Olympus Corporation, Tokyo, Japan).

### Flow cytometry and cell cycle analysis

Cells were washed in ice-cold PBS and fixed with 80% ethanol for 2 h. The fixed cells were then incubated in FxCycle PI/RNase staining solution (Thermo Fisher Scientific) at room temperature for 30 min. Cell cycle analysis was performed using a Beckman Coulter flow cytometer (Beckman Coulter, Brea, CA, USA). The distribution of cells at each cell phase was measured on computerized integrated optical density histograms.

### Western blotting analysis

Protein samples were harvested from cell lysates and the concentration of the concentration of total protein was measured with a BCA Protein assay kit (Beyotime Biotechnology, Bejing, China). Equal amount of protein extracts was separated by 10% sodium dodecyl sulphate-polyacrylamide gels (SDS-PAGE) and then transferred to polyvinylidene difluoride (PVDF) membranes (Thermo Fisher Scientific). The membranes were blocked for 1 h in in PBS containing 5% nonfat milk at room temperature and then incubated with primary antibodies at 4 °C overnight: rabbit anti-TNFAIP8 antibody (1:500 dilution; ab166804, Abcam, MA, USA), rabbit anti-p53 antibody (1:500 dilution; ab131442), rabbit anti-E-cadherin antibody (1:500 dilution; ab15148), rabbit anti-N-cadherin antibody (1:500 dilution; ab18203), rabbit-anti-Vimentin antibody (1:1000 dilution; ab92547), rabbit anti-Snail antibody (1:500 dilution; ab82846) and rabbit anti-β-actin antibody (1:1000 dilution; #4970, Cell Signaling Technology, MA, USA). After being washed with PBS, the membrane was probed with corresponding goat anti-rabbit IgG H&L secondary antibodies for 1 h (1:2000 dilution; #ab6721). Blots were visualized using enhanced chemiluminescence (Thermo Fisher Scientific) and brand intensity was measured by densitometry using Quantity One software (Bio-Rad, Hercules, CA, USA).

### Histopathological analysis

Paraffin-embedded breast cancer tissues were routinely fixed with 10% formalin and embedded in paraffin, and were sliced into 5-μm-thick sections. After being baked at 60 °C for 2 h, the tissues were incubated with xylene for deparaffinization and decreasing concentrations of ethanol for rehydration. Hemotoxylin solution and eosin solustion were used in turn for hematoxylin–eosin staining (HE). For immunohistochemistry analysis, antigen retrieval was conducted in Target Retrieval Solution, pH9 (Dako, Carpinteria, CA, USA), and 4% hydrogen peroxide H_2_O_2_ was used to block endogenous tissue peroxidases for 10 min. Tissue slides were treated with normal goat serum to avoid nonspecific staining and incubated overnight at 4 °C with anti-TNFAIP8 antibody (#ab166804, Abcam, 1:100). Tissue slides were them incubated with goat anti-rabbit IgG H&L as a secondary antibody at 37 °C for 1 h. Quantification of protein levels was assessed by proportion of positive immunostaining cells.

### RNA pull-down

P53 transcription was performed using the T7 RNA promoter (Roche, Indianapolis, IN, USA) in vitro, then by using the RNeasy Plus Mini Kit (Qiagen, Germantown, MD, USA). Purified RNAs were biotin-labeled with a biotin RNA labeling mix (Roche, Indianapolis, IN, USA). Biotin-labeled RNAs were added into cell lysate and subsequently incubated at room temperature for 1 h, followed by addition of washed streptavidin-agarose beads. After washed four times, the beads were boiled for 30 min, and the eluted proteins were checked with western blot analysis.

### Dual-luciferase reporter assay

For the dual-luciferase reporter assay, the 3′UTR of TNFAIP8 was amplified by PCR and inserted into downstream of the firefly luciferase reporter gene in the pGL3-Basic vector (GenePharma). BT-549 and MDA-MB-231 cells were seeded in 24-well plates and transiently transfected with appropriate pcDNA3.1 and luciferase plasmids using lipofectamine 3000 (Invitrogen, Carlsbad, CA, USA). After 48 h transfection, cell lysates were harvested, and the luciferase activities were measured by the Luc-Pair™ Duo-Luciferase Assay Kit (Genecopoeia, Guangzhou, China). *Firefly* luciferase activities were normalized to *Renilla* luciferase activities. All experiments were repeated three times.

### Animal experiments

Four- to five-week-old male BALB/c nude mice were purchased from Hunan SJA Laboratory Animal company. Stable BT-549 and MDA-MB-231 cells (2 × 10^6^ per 100 PBS μl) transfected with lncRNA or TNFAIP8 siRNAs were subcutaneously injected into mice and injected. The width and length of the formed tumors was examined every 5 days after injection. Tumor volumes were calculated using the following formula: tumor volume = width^2^ × length × 0.5. At 4-week post injection, the mice were killed by cervical dislocation, and the tumors were excised and photographed. The tumor tissues and lymph nodes were subsequently harvested, embedded, fixed, and prepared for histopathological staining and western blot analyses. A lymph node set was considered macroscopically invaded if its total mass exceeded 30 mg.

## Results

### LncRNA H19 and TNFAIP8 expression is up-regulated in breast cancer tissues and cell lines, especially in TNBC cell lines

**Fig. 1:**
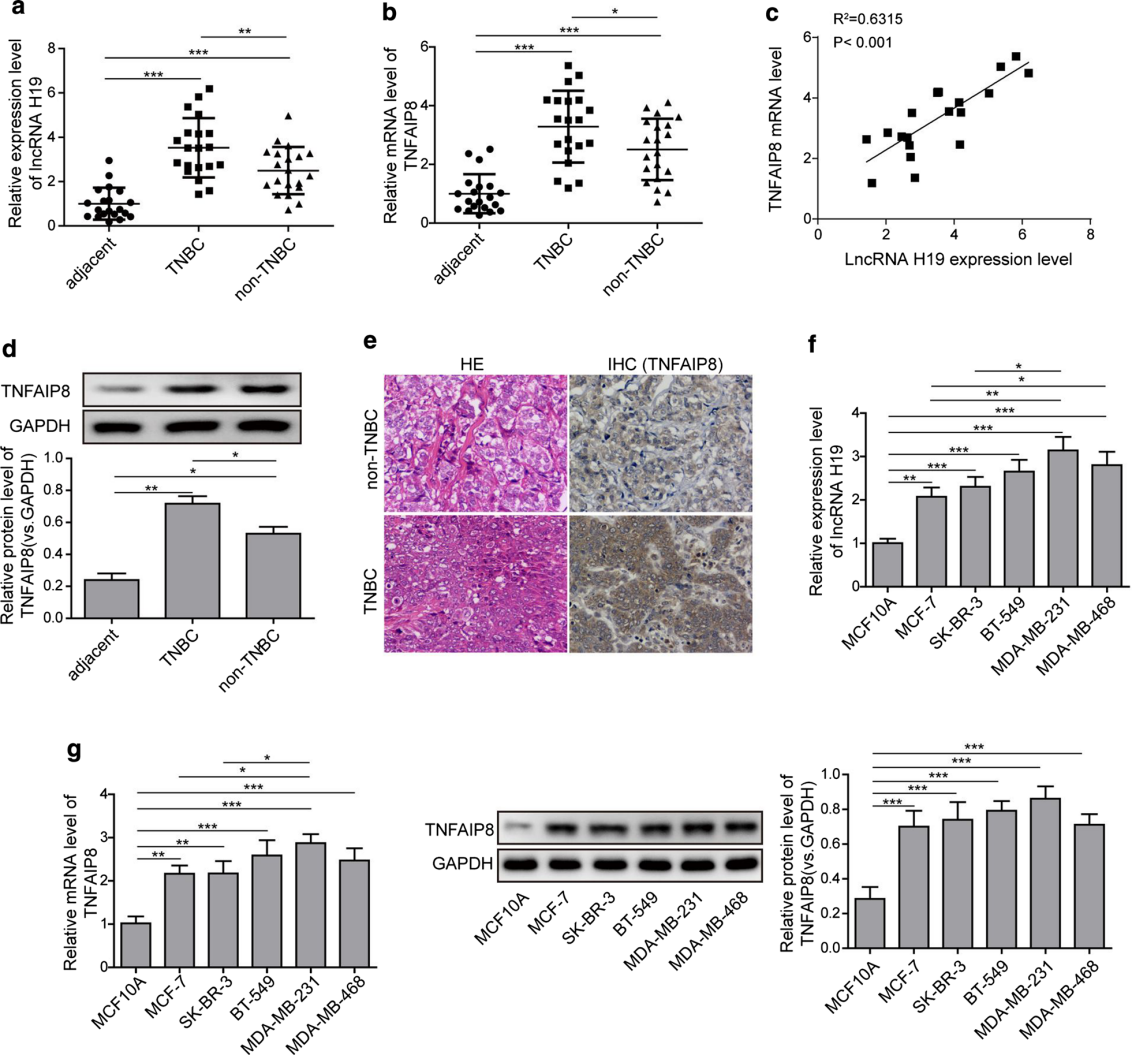
Expression patterns of lncRNA H19 and TNFAIP8 in breast cancer tissues and cell lines. **a** The expression levels of lncRNA H19 in breast cancer tissues (non-TNBC and TNBC) and adjacent normal tissues detected by qRT-PCR. **b** The mRNA expression and protein levels of TNFAIP8 in breast cancer tissues (non-TNBC and TNBC) and adjacent normal tissues detected by qRT-PRC and Western blotting analysis. **c** A correlation analysis between the levels of lncRNA H19 and TNFAIP8 mRNA in triple-negative breast cancer (TNBC) samples. **d** Immunohistochemical (IHC) staining of TNFAIP8 in breast cancer tissues (non-TNBC and TNBC) and adjacent normal tissues. Original magnification, 200 × . **e** The protein levels of TNFAIP8 in breast cancer tissues (non-TNBC and TNBC) and adjacent normal tissues detected by Western blotting analysis. **f** The expression levels of lncRNA H19 in diverse breast cancer cell as compared with the human breast epithelial cell line MCF10A detected by qRT-PRC. **g** The expression and protein levels of TNFAIP8 in diverse breast cancer cell as compared with MCF10A detected by qRT-PRC and Western blotting analysis. Measurements were carried out in triplicate, and experiments were repeated three times. Data are presented as mean ± SD. * p < 0.05, ** p < 0.01 and *** p < 0.001

We first determined the expression of lncRNA H19 and TNFAIP8 in breast cancer tissues and cells by qRT-PCR and western blotting analysis. Both lncRNA H19 and TNFAIP8 were significantly up-regulated in tumor tissues from TNBC compared with that in tumor tissues from non-TNBC and adjacent normal tissues (Fig. 1a, b, d). Furthermore, there was a significant positive correlation between the expression levels of H19 and TNFAIP8 in TNBC samples (Fig. 1c). IHC analysis further confirmed the elevated TNFAIP8 protein in TNBC tissues (Fig. 1e). Moreover, the enhanced expression of lncRNA H19 and TNFAIP8 was also evident in serial breast cancer cell lines, particularly in TNBC cells (Fig. 1f, g). As lncRNA H19 and TNFAIP8 mRNA levels were much more abundant in BT-549 and MDA-MB-231 cells than in other cell lines (Fig. 1f, g), so these two human TNBC cell lines were selected for the following experiments. These data confirmed both the involvements of lncRNA H19 and TNFAIP8 in the progression of breast cancer, with the special regard to TNBC.

### Knockdown of TNFAIP8 inhibits cell proliferation, migration and invasion of breast cancer

**Fig. 2:**
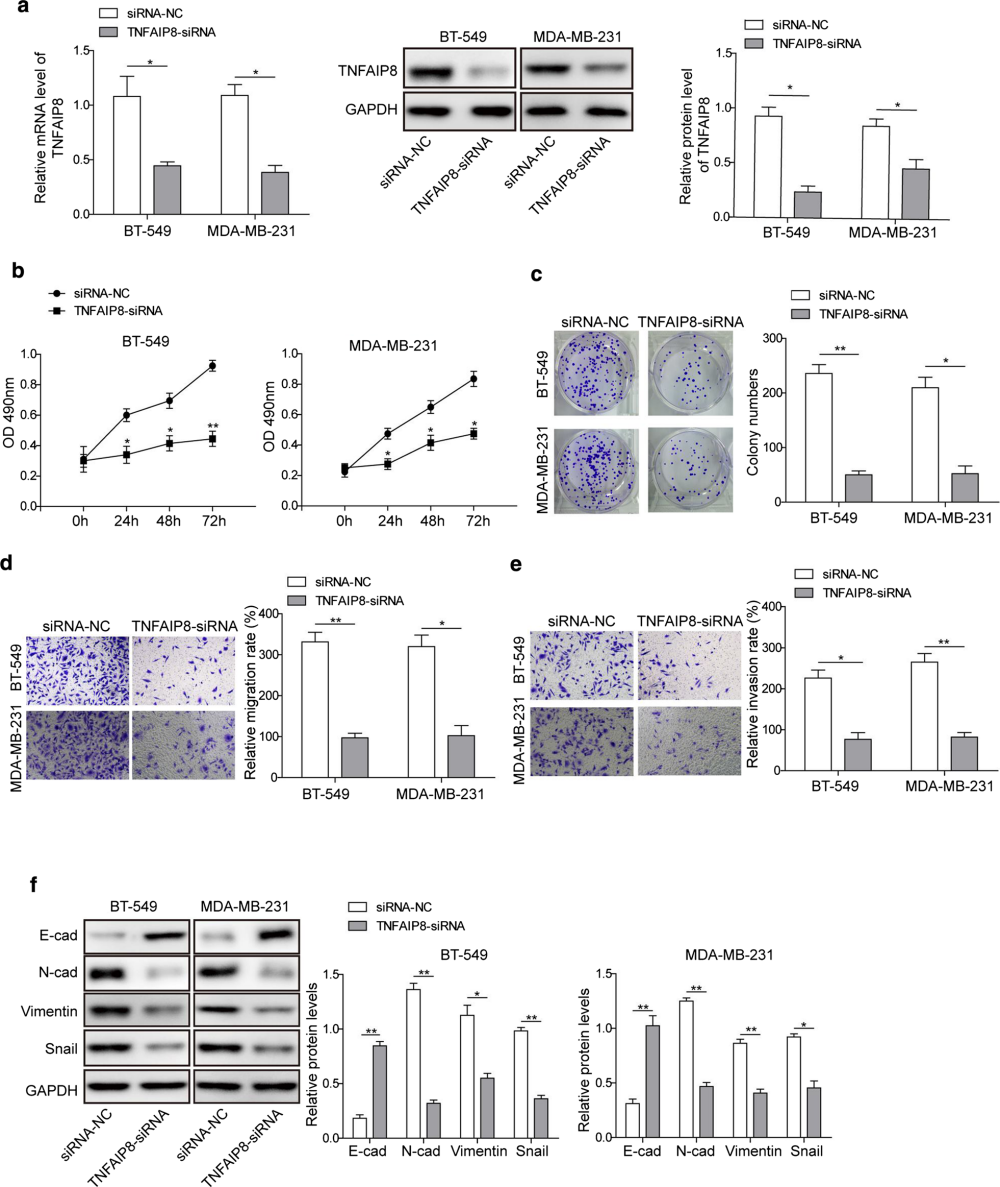
Effect of TNFAIP8 depletion on proliferation, migration and invasion of breast cancer cells. **a** Inhibition of TNFAIP8 reduced the expression and protein levels of TNFAIP8 in BT-459 and MDA-MB-231 cells detected by qRT-PCR and Western blotting analyses. **b** Inhibition of TNFAIP8 inhibited proliferation of BT-459 and MDA-MB-231 cells detected by CCK-8 assay. **c** Inhibition of TNFAIP8 reduced clonogenicity of BT-459 and MDA-MB-231 cells detected by colony formation assay. **d**–**e** Inhibition of TNFAIP8 repressed migration and invasion of BT-459 and MDA-MB-231 cells detected by Transwell assay. **f** Protein levels of N-cadherin, Vimentin and Snail, and E-cadherin in BT-459 and MDA-MB-231 cells with treatment of TNFAIP8-siRNA detected by Western blotting analysis. Measurements were carried out in triplicate, and experiments were repeated three times. Data are presented as mean ± SD. * p < 0.05 and ** p < 0.01

Given that TNFAIP8 is overexpressed in breast cancer cells, we then wanted to determine whether inhibition of TNFAIP8 by siRNA in BT-549 and MDA-MB-231 cells could influence cell proliferation, migration, and invasion. qRT-PCR and Western blotting analyses confirmed the efficient siRNA-mediated knockdown of TNFAIP8 (Fig. 2a). Results from CCK-8 assay indicated that the proliferation ability of BT-549 and MDA-MB-231 cells with TNFAIP8 knockdown was markedly lower than that in the control group (Fig. 2b). In addition, inhibiting the expression of TNFAIP8 markedly repressed cell colony-forming activity (Fig. 2c) and quantification analysis through transwell assays further showed a dramatic reduction in migration and invasive abilities of BT-459 and MDA-MB-231 cells after TNFAIP8 inhibition (Fig. 2d, e). Moreover, N-cadherin, vimentin, and snail protein was downregulated, and E-cadherin protein was upregulated in BT-459 and MDA-MB-231 cells after treatment with TNFAIP8-siRNA (Fig. 2f). Collectively, these data indicate that TFNAIP8 may promote the cell growth and EMT process in breast cancer cells.

### LncRNA H19 promotes cell proliferation, migration, and invasion and leads to cell cycle arrest of breast cancer through upregulation of TNFAIP8

**Fig. 3:**
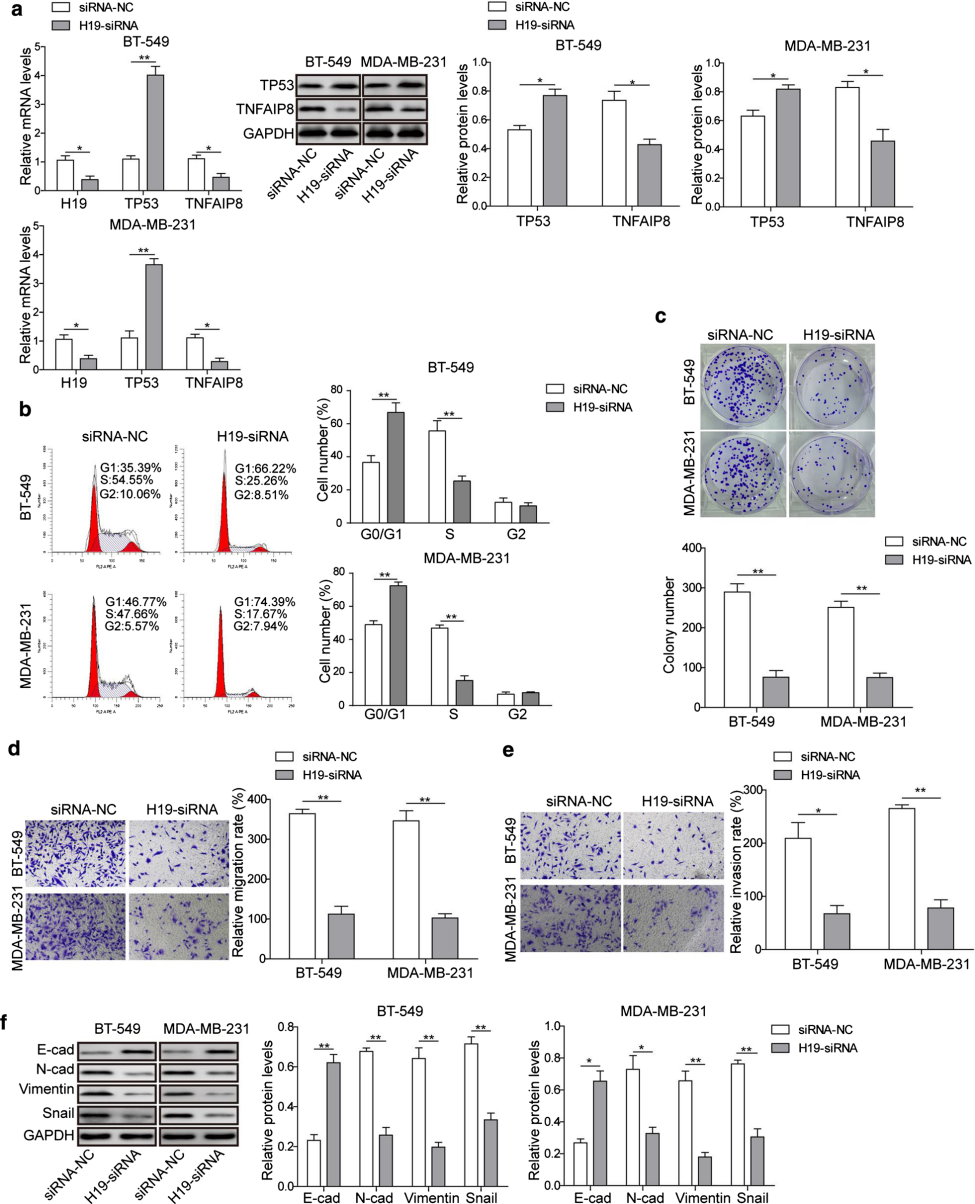
Effect of lncRNA H19 depletion on proliferation, migration, invasion and cell cycle arrest of breast cancer cells. **a** The expression levels of lncRNA H19, p53, and TNFAIP8 (left) and the protein levels of p53 and TNFAIP8 (right) in BT-549 and MDA-MB-231 cells treated with H19-siRNA detected by qRT-PCR and Western blotting analyses. **b** Cell cycle profiles of BT-549 and MDA-MB-231 cells treated with H19-siRNA detected by flow cytometry assay. **c** Inhibition of lncRNA H19 reduced clonogenicity of BT-459 and MDA-MB-231 cells detected by colony formation assay. **d**–**e** Inhibition of lncRNA H19 repressed migration and invasion of BT-459 and MDA-MB-231 cells detected by Transwell assay. **f** Protein levels of N-cadherin, Vimentin and Snail, and E-cadherin in BT-459 and MDA-MB-231 cells with treatment of H19-siRNA detected by Western blotting analysis. Measurements were carried out in triplicate, and experiments were repeated three times. Data are presented as mean ± SD. * p < 0.05 and ** p < 0.01

We next examined the effect of lncRNA H19 inhibition by siRNA on cell activities including proliferation, migration, invasion and cell cycle arrest. Real-time PCR analysis confirmed the efficient siRNA-mediated knockdown of lncRNA H19 (Fig. 3a). Silencing of lncRNA H19 did indeed result in a significant increase in p53 transcript levels in both BT-459 and MDA-MB-231 cells, accompanied by a concomitant decrease in TNFAIP8 transcript levels (Fig. 3a). Western blotting analysis further confirmed the decreased p53 and TNFAIP8 protein in BT-459 and MDA-MB-231 cells after lncRNA H19 silencing (Fig. 3a). Moreover, a cell cycle distribution analysis revealed that loss of lncRNA H19 facilitated cell cycle arrest at G0/G1, as reflected by an obvious increase in percentage of cells in the G0/G1 phase and an remarkable reduction in the S phase (Fig. 3b). As expected, inhibiting the expression of lncRNA H19 markedly repressed cell colony-forming activity, migration and invasion in both BT-549 and MDA-MB-231 cells (Fig. 3c–e), to the extent similar to that observed after TNFAIP8 depletion. Similarly, N-cadherin, vimentin, and snail protein was significantly reduced, and E-cadherin protein was significantly enhanced following lncRNA H19 silencing (Fig. 3f). Taken together, these findings suggest that lncRNA H19 promoted cell growth and EMT process in breast cancer cells, possibly through the positive regulation of TNFAIP8.

### P53 acts as a transcriptional inhibitor of TNFAIP8

**Fig. 4:**
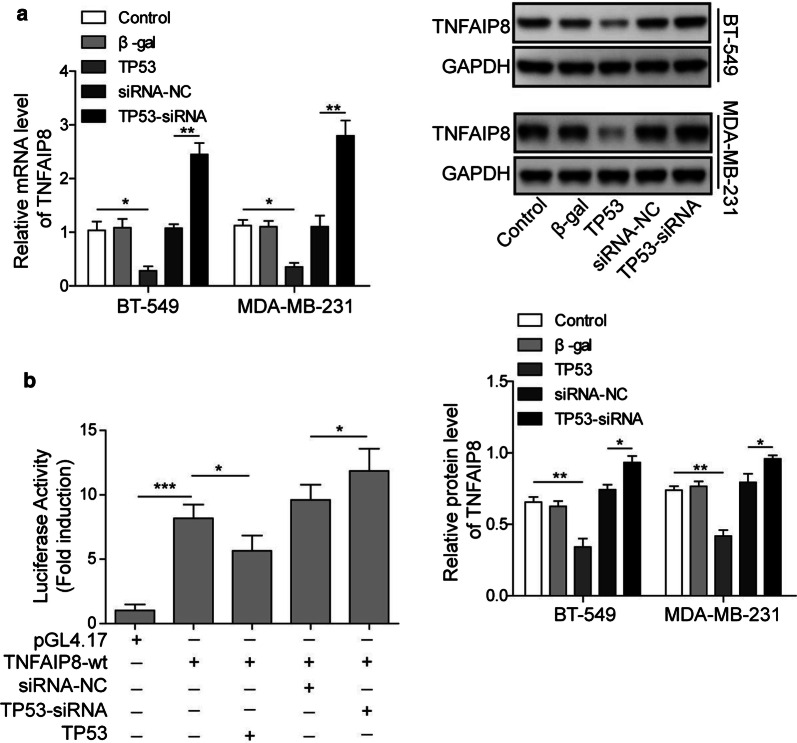
P53 acts as a transcriptional inhibitor of TNFAIP8. **a** The mRNA (upper) and protein (lower) levels of TNFAIP8 in BT-549 and MDA-MB-231 cells co-transfected with control, beta-gal, p53, siRNA-NC, or p53-siRNA. **b** Relative luciferase activity in pGL3-TNFAIP8-WT-treated BT-549 and MDA-MB-231 cells co-transfected with pGL4.17, siRNA-NC, p53-siRNA, or p53, respectively. Measurements were carried out in triplicate, and experiments were repeated three times. Data are presented as mean ± SD. * p < 0.05, ** p < 0.01and *** p < 0.001

Previous study has demonstrated that TNFAIP8 variant 2 in MCF-7 cells is a p53-regulated gene product that reciprocally interacts with p53 to promote tumorigenesis [26]. As expected, overexpression of p53 inhibited both the expression and protein levels of TNFAIP8, whereas loss of p53 had an opposite effect (Fig. 4a), indicating that p53 negatively regulates TNFAIP8 stability at translation level. We then performed dual-luciferase reporter assay to validate whether TNFAIP8 is a target of p53. The promotor sequences of TNFAIP8 was then inserted to the upstream of luciferase coding region (pGL3-TNFAIP8-promotor) and then transfected into BT-549 and MDA-MB-231 cells, which were co-transfected with plasmids encoding p53 or p53-siRNA in parallel to control plasmid. The Results showed that p53 significantly reduced the luciferase activities in pGL3-TNFAIP8-WT-treated BT-549 and MDA-MB-231 cells, while inhibition of p53 by siRNA significantly increased the luciferase activities in pGL3-TNFAIP8-WT-treated cells (Fig. 4b). These data indicated that P53 functions as a transcriptional inhibitor of TNFAIP8.

### LncRNA H19 functions as a competitive inhibitor of p53 and abrogates its transcriptional inhibition of TNFAIP8

**Fig. 5:**
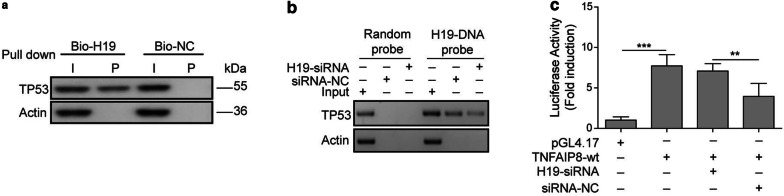
LncRNA H19 functions as a competitive inhibitor of p53 and abrogates its transcriptional inhibition of TNFAIP8. **a** The targeting relations of lncRNA H19 and p53 were assessed by RNA pull-down assay. Endogenous p53 enrichment was carried out specifically in lncRNA H19 probe detection as compared with the control group. LncRNA H19 was incubated with nuclear extracts, and p53 protein was measured by western blotting analysis. **b** The targeting relations of lncRNA H19 and p53 were confirmed by RNA pull-down assay. Endogenous p53 was attenuated specifically in lncRNA H19 probe detection treated with H19-siRNA compared with control group. **c** Relative luciferase activity in pGL3-TNFAIP8-WT-treated BT-549 and MDA-MB-231 cells co-transfected with pGL4.17, siRNA-NC, or H19-siRNA, respectively. Measurements were carried out in triplicate, and experiments were repeated three times. Data are presented as mean ± SD. ** p < 0.01 and *** p < 0.001

Previous studies have proved that lncRNA H19 could interact with p53 protein and negatively regulate its function in gastric- and bladder cancer cells [20, 21]. Our qRT-PCR assay also showed p53 expression was significant higher in breast cancer cells after lncRNA H19 knockdown (Fig. 3a). We then conducted RNA pull-down assay to clarify the association between lncRNA H19 and p53. A significant enrichment of p53 was observed in lncRNA H19 probe detection compared with control group (Fig. 5a). In addition, endogenous p53 was repressed in lncRNA H19 probe treated with H19-siRNA (Fig. 5b), suggesting that lncRNA H19 is a direct inhibitory target of p53. Dual-luciferase reporter assay was subsequently carried out to validate the effect of lncRNA H19 on TNFAIP8. Inhibition of lncRNA H19 by siRNA did indeed significantly reduce the luciferase activity in pGL3-TNFAIP8-promotor-treated BT-549 and MDA-MB-231 cells (Fig. 5c). Taken together, these findings suggested that lncRNA H19 may play its pro-tumor role through protecting TNFAIP8 from downregulation by p53.

### Knockdown of lncRNA H19 inhibits xenograft growth via regulating p53/TNFAIP8 axis in vivo

**Fig. 6:**
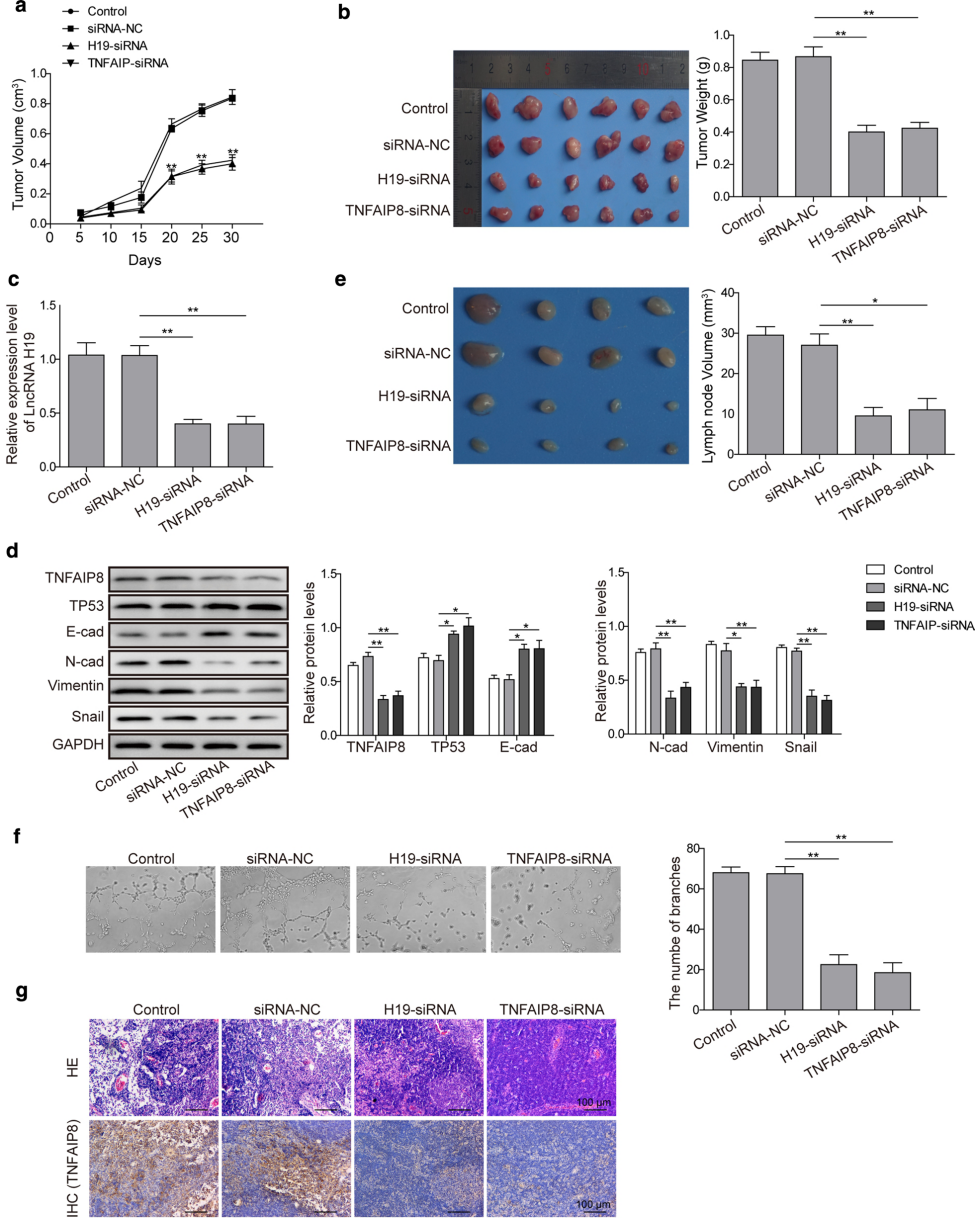
LncRNA H19 promotes xenograft growth via regulating p53/TNFAIP8 axis in vivo. **a** Tumor volume of the xenografts was remarkably inhibited by depletion of lncRNA H19 or TNFAIP 8. **b** The xenograft tumors were significantly depressed (left) and tumor weight was remarkably reduced by lncRNA H19 or TNFAIP8 depletion. **c** Silencing of lncRNA H19 or TNFAIP8 significantly reduced the expression levels of lncRNA H19 in vivo detected by qRT-PCR. **d** The protein levels of TNFAIP8, p53, and EMT markers in the xenografted tissues detected by Western blotting analyses. **e** The lymph node metastases were significantly inhibited by lncRNA H19 or TNFAIP8 depletion. **f** Tumor lymph angiogenesis was significantly reduced by lncRNA H19 or TNFAIP8 depletion. **g** Histopathological analysis (HE staining and TNFAIP8 IHC staining) of the lymph node samples isolated from xenografted mice. Original magnification, 100× . Measurements were carried out in triplicate, and experiments were repeated three times. Data are presented as mean ± SD. *p < 0.05 and **p < 0.01

Finally, we explored the molecular effect of lncRNA and TNFAIP8 inhibition on tumor growth in vivo. BT-549 and MDA-MB-231 cells transfected with H19-siRNA or si-TNFAIP8 were used in a nude mice xenograft model. Thirty days after subcutaneous injection, the tumors formed in H19-siRNA and si-TNFAIP8 groups were substantially smaller than those in the negative control group, as evidenced by a dramatic decrease in tumor volume and tumor weight (Fig. 6a, b). The mRNA expression of lncRNA H19 was markedly reduced in tumor-bearing mice after lncRNA H19 or TNFAIP8 knockdown (Fig. 6c), which likely contributed to its weaker ability to promote tumor growth. In addition, TNFAIP8 protein was downregulated, and p53 protein was upregulated in tumor-bearing mice treated with lncRNA H19 or TNFAIP8 siRNA (Fig. 6d). This was accompanied by increased E-cadherin protein and decreased N-cadherin, Vimentin, and Snail protein (Fig. 6d). Moreover, inhibition of lncRNA H19 or TNFAPI8 significantly reduced lymph node metastases, as evidenced by a greater than 50% decrease in lymph node volume in mice bearing lncRNA-siRNA or TNFAIP8-siRNA-expressing tumors (Fig. 6e). Notably, tumor lymph angiogenesis was also significantly repressed after lncRNA H19 or TNFAIP8 knockdown (Fig. 6f). Pathological staining analysis of the lymph node samples using HE staining and TNFAIP8 IHC staining also revealed that suppression of lncRNA H19 reduced significantly vacuole and necrosis lesions in lymph node tissues induced by tumor cells, as well as the expression level of TNFAIP8 protein (Fig. 6g). These results demonstrate that knockdown of lncRNA H19 inhibits tumorigenesis and metastases of breast cancer cells through p53/TNFAIP8 pathway in vivo.

## Discussion

Our findings demonstrate that lncRNA H19 was more abundant in breast cancer cells, particularly in TNBC cells, than in normal mammary epithelial cells. Furthermore, lncRNA H19 functioned as a competitive inhibitor of p53 and abolished its transcriptional repressive effect on TNFAIP8 promotor. The H19/p53/TNFAIP8 axis promoted a series of tumor cell activities including proliferation, migration, invasion, EMT and metastasis both in vitro *and* in vivo, whereas silencing of lncRNA H19 exerted suppressive effects on these cell activities in breast cancer.

Currently, a rising number of studies have managed to investigate the association between lncRNAs and breast cancer. LncRNAs have dual effects in BC metastasis by regulating invasion, migration, and distant metastasis of breast cancer cells [27]. In addition, lncRNAs have critical regulatory effects in the stemness and angiogenesis of breast cancer [28]. LncRNA H19 has been identified to be functionally associated with many biological processes, such as cell proliferation, invasion, and apoptosis of tumors, with its crucial role in tumorigenesis breast cancer has been increasingly recognized [28]. In particular, enhanced lncRNA H19 expression was closely linked with poor disease-free survival and overall survival, especially in patients with TNBC [8]. Our findings confirmed the protumor effects of lncRNA H19 in breast cancer cells, in which depletion of lncRNA H19 significantly suppressed cell proliferation, migration and invasion.

LncRNA H19 can exert its carcinogenesis effects through diverse mechanisms. It can promote tumorigenesis of breast cancer cells [11] via serving as a myc up-regulated gene. It can also regulate tumor progression through functioning as a miRNA sponge, for instance, to modulate the let-7 miRNA family members [12]. Moreover, it has been reported that H19 might serve as a gene-expression regulator at the post-transcriptional level through acting as a precursor of miR-675 [13]. Notably, our data herein suggest a novel model to depict lncRNA H19-p53 interplay during carcinogenesis and metastasis of breast cancer, which is consistent with previous reports [20].

The tumor suppressor p53 regulates downstream genes that have been implicated in cell cycle arrest and apoptosis [29]. Importantly, lncRNA H19 locus has been placed at the core of cell cycle control, especially in the transition from G1 to S phase [9]. Enhanced lncRNA H19 expression in breast cancer cells lines induces G1/S cell cycle transition while lncRNA H19 depletion blocks S-phase entry and proliferation [30, 31]. Moreover, lncRNA H19 derived miR-675 plays an important role in repressing the expression of p53 and p53-dependent protein in bladder cancer cells [21]. This raised the possibility that the G0/G1 cell cycle arrest in breast cancer induced by lncRNA H19 depletion in our study may be due to the repression of p53 function. And for the first time, we discovered that lncRNA H19 is a target gene of p53 in breast cancer.

Furthermore, we identify TNFAIP8 as a tumor-promoter that are required for several hallmark traits of breast cancer cells, such as maintenance of proliferative signaling and promotions of lymph node metastasis. TNFAIP8 can exert an anti-apoptotic or a pro-apoptotic function, depending on the cellular context [32, 33]. Typically, TNFAIP8 is a direct target of the transcription factor nuclear factor-κB (NF-κB), which is potently activated by the cytokine tumor necrosis factor [34]. In colon cancer, mutant k120 p53 activated TNFAIP8, which in turn suppressed caspase 8 to inhibit cell apoptosis [35]. In non-small cell lung cancer, TNFAIP8 negatively regulates p53 stability at the post-translational level and promotes cell growth by stimulating G1-specific cyclin D1, resulting in the G1 to S cell cycle transition [36]. And our findings highlight the reciprocal regulatory interaction between TNFAIP8 and p53 in breast cancer, consistent with previous reports [26, 33]. Importantly, our lncRNA H19 and TNFAIP8-silencing studies in steady-state BT-459 and MDA-MB-231 cells promoted progression and metastasis of breast cancer cells, and we demonstrated that enhanced lncRNA H19 expression abrogated the repressive effect of p53 on TNFAIP8 through competitively binding to p53, and this in turn induced EMT and metastasis of breast cancer cells.

EMT plays a crucial role in the development of tumor metastasis [37]. Emerging evidence has highlighted the crucial role of lncRNA H19 in regulating EMT-MET process [10]. Studies from several cancers show that lncRNA H19 promotes EMT by downregulating E-cadherin through diverse molecular mechanisms, such as stimulating the Wnt/β-catenin signaling pathway [38], and acting as ceRNA for miRNAs [9, 39]. In a spontaneous metastatic mouse model of breast cancer, lncRNA H19 has been found to regulate EMT and MET by differentially functioning as a sponge for the microRNA miR-200b/b and let-7b [40]. Consistently, we uncovered that lncRNA H19 can cause induction of EMT through a mechanism involving reduced E-cadherin expression. Importantly, using xenograft MDA-MB-231 tumor-bearing mice, we found that lncRNA H19 was also involved in promoting cancer metastasis. Further studies into the in vivo metastasis in various metastasis models are warranted to clarify lncRNA H19′s function in different breast cancer subtypes and in different stages of cancer progression.

## Conclusions

We demonstrated that the tumor-promoting role of lncRNA H19 in breast cancer, especially TNBC, which is in line with many previous studies. And we displayed a novel mechanism by which lncRNA H19 regulates EMT and MET in breast cancer development and progression through sequestering p53 and upregulating TNFAIP8. Blockage of the lncRNA H19/p53/TNFAIP8 axis may be a promising approach to inhibit EMT and metastasis of breast cancer. These findings thus provide insights into further functional, diagnostic, and therapeutic research of lncRNA H19 in breast cancer.

## Data Availability

All data generated or analysed during this study are included in this published article.
